# Proposed Definitions and Clinical Recommendations for the Management of Weight Recurrence, Partial Response, and Nonresponse Following Metabolic and Bariatric Surgery

**DOI:** 10.1007/s11695-025-08359-6

**Published:** 2025-12-01

**Authors:** Saniea F. Majid, Shushmita Ahmed, Sue Benson-Davies, David Voellinger, Matthew Davis, Saad Ajmal, Franchell Richard Hamilton, Mohamed Ali, Stephen Archer

**Affiliations:** 1 Weight Loss and Wellness Center, Springfield, New Jersey, United States; 2 Department of Surgery University of California, Davis Health, Sacramento, CA Center for Alimenatry and Metabolic Science, University of California, Davis School of Medicine, Sacramento CA, University of California, Davis, Sacramento, United States; 3 Department of Surgery, Sanford School of Medicine, University of South Dakota., University of South Dakota, Vermillion, United States; 4 Novant Health Bariatric Solutions, Charlotte, North Carolina, United States; 5 University of Tennessee Health Science Center, Memphis, United States; 6 CAMC Weight Loss Center, Charleston Area Medical Center, Charleston, United States; 7 NeuroSwitch Weight Loss Institute, Northlake, Texas, United States; 8 Summit Medical Group, St. Charles Medical Center, Bend, United States

## Abstract

**Background:**

Weight recurrence (WR), partial response, and nonresponse following metabolic and bariatric surgery (MBS) remain poorly defined. This lack of standardization hinders timely diagnosis, clinical management, and outcomes research. Moreover, no consensus guidelines currently exist to guide providers in treating these conditions.

**Objectives:**

To evaluate current practices in managing WR, partial response, and nonresponse after MBS; to propose standardized definitions; and to offer evidence-informed clinical recommendations for their identification and treatment.

**Methods:**

A systematic literature review was conducted using terms related to WR, partial response, and nonresponse following primary MBS. The review focused on management strategies, clinical guidelines, and algorithms addressing these conditions.

**Results:**

A total of 119 articles published between 2020 and 2023 were included. Of these, 52% were retrospective, 31% prospective or observational, and 7.6% randomized controlled trials. Nearly half (45%) did not define WR or distinguish partial and nonresponse. Among studies that defined WR, definitions varied widely. Clinical management strategies were heterogeneous, and only 2.5% proposed structured treatment algorithms.

**Conclusion:**

We propose standardized definitions for WR, partial response, and nonresponse after MBS, along with tailored management algorithms. These recommendations aim to support a unified clinical approach and facilitate research in this complex and evolving area of bariatric care.

## Introduction

Metabolic and bariatric surgery (MBS) remains the most effective and durable treatment for obesity. In addition to promoting weight loss, MBS modifies the natural history of the disease, leading to reduced comorbidity progression and improved cardiovascular and all-cause morbidity and mortality [1–4]. On average, patients reach their nadir weight within 24 months postoperatively, and 50% maintain weight loss at five years [5, 6].

Weight recurrence (WR) is not uncommon, with reported rates ranging from 9% to 91%, depending on the definition used [7]. WR can contribute to comorbidity recurrence, increased healthcare costs, and diminished quality of life and emotional well-being [8–10]. Nevertheless, most patients retain significant health benefits compared to their preoperative status, underscoring that WR reflects the chronic nature of obesity rather than surgical failure. Importantly, WR is often conflated with partial response and nonresponse—distinct biological phenomena with unique pathophysiologies. As awareness of WR increases and both surgical and nonsurgical treatment options expand (e.g., revisional surgery and anti-obesity medications), clinical guidelines are needed to support timely identification and optimized management.

The first step in managing WR is establishing a standardized definition. Currently, no consensus exists on what constitutes clinically significant WR after MBS [11]. Definitions vary widely by time frame, weight loss metric, degree of weight regain, and study design [12, 13]. Parameters may include nadir weight, percent total weight loss (TWL), or excess weight loss (EWL), further contributing to inconsistency. This heterogeneity underscores the urgent need for unified definitions to guide diagnosis and management.

The American Society for Metabolic and Bariatric Surgery (ASMBS) Postoperative Weight Recurrence (POWER) Task Force previously highlighted the absence of a consensus definition for WR as a critical barrier to advancing research and care [14]. Their review emphasized two key issues: inconsistent terminology for postoperative weight changes and the lack of an agreed-upon WR definition. Given that obesity is a chronic, relapsing disease requiring multimodal care, the task force recommended an oncologic model, advocating for the terms “weight recurrence” rather than “weight regain” and “partial/nonresponse” in place of stigmatizing phrases like “weight loss failure.” The group also called for a coordinated, evidence-based effort to define clinically significant WR.

The aim of this paper is to propose evidence-informed definitions for WR, partial response, and nonresponse following MBS, and to offer practical clinical recommendations for their management. We conducted a targeted literature review to assess current practices and identify gaps in care. Through this work, we hope to modernize terminology and establish a standardized clinical framework for these increasingly recognized conditions.

## Methods

We conducted a literature search of peer-reviewed articles published between 2020 and 2023 using PubMed. The following keywords were used: “weight regain and bariatric,” “insufficient weight loss and bariatric,” and “weight recidivism and bariatric.” Although “weight recurrence” is the preferred term, its recent adoption required the inclusion of legacy terminology to capture all relevant studies. A total of 149 articles were initially identified.

Articles were included if they met the following criteria:

- Published in English.
- Peer-reviewed.
- Addressed WR following primary bariatric procedures.
- Contained clinical guidelines, management algorithms, or treatment recommendations.

We excluded studies focusing on surgical gastric plication or endoscopic therapies such as intragastric balloon placement.

A total of 119 articles met inclusion criteria and underwent full review. We performed a thematic analysis of these studies, focusing on definitions of WR, partial response, and nonresponse; reported management strategies; and proposed treatment algorithms.

## Results

Of the 119 studies included, 62 (52%) were retrospective reviews, 37 (31%) were prospective or observational studies, and 9 (7.6%) were randomized controlled trials. The remaining studies comprised 3 systematic reviews, 3 qualitative studies, 2 meta-analyses, 1 Delphi consensus, and 1 cross-sectional intervention. Notably, most studies focused on interventions for weight-related outcomes rather than defining WR, partial response, or nonresponse.

Definitions of WR varied widely (Table 1). Criteria ranged from nadir weight to percent total weight loss (TWL) or excess weight loss (EWL) [15–19]. Fifty-four studies (45.4%) did not define WR. Furthermore, partial and nonresponse were often conflated with WR, despite being distinct conditions [20]. Recent studies have employed percent maximum weight loss as a surrogate for WR, while arbitrary cutoffs (e.g., < 50% EWL) were frequently used to define surgical “success” or “failure”, though none were supported by outcomes data [21, 22].

**Table 1:** Variability of terminology and definitions across studies. Of note, only 55% of studies reviewed included a definition for weight recurrence (WR)

Terminology	Definition
Weight Recurrence	> 20% increase from nadir weight 10% Weight regain Inability to maintain %50 EWL^a^ after 24 months
Percent Maximum Weight Loss	[100*(post-nadir weight – nadir weight)]/(pre-surgery weight – nadir weight)
Insufficient Weight Loss	< 20% TWL^b^
Weight Loss Failure	< 50% EWL after 24 months

^a^ *EWL* Excess weight loss

^b^ *TWL* Total body weight loss

Only 64 studies (54%) discussed clinical management strategies for WR. Recommendations varied based on the discipline of the reporting authors. Dietitians emphasized behavioral and lifestyle modification, while surgeons favored multimodal approaches incorporating pharmacotherapy, endoscopy, and surgical revision. Only 3 studies (2.5%) proposed structured treatment algorithms. These generally followed a stepwise model, beginning with dietary and behavioral interventions, progressing to pharmacotherapy, and, if needed, escalating to endoscopic or surgical revision [18, 23–32] (Table 2).

**Table 2:** Variability of intervention and study design for weight recurrence, and partial/nonrecurrence as found in literature review

Intervention	Study design
Lifestyle Interventions	Lifestyle, behavioral, and dietary modifications, or surgical revision
	Lifestyle, weight-loss pharmacotherapy, and non-weight-loss pharmacotherapy
	Binge Eating Scale
Endoscopic Management	Intra-gastric balloon placement
	Endoscopic management of sleeve stenosis
	Endoscopic sleeve gastroplasty +/- weight loss medications
	Endoscopic outlet reduction
	Transoral outlet reduction (TORe)
Pharmacotherapy	Liraglutide 3.0 mg
	Topiramate, topiramate + sibutramine, sibutramine only, orlistat
Surgery	Abdominoplasty
	Conversion of LSG to RYGB or OAGB

A majority of studies (68%) identified significant knowledge gaps in the literature regarding the management of WR (Table 3). Contributing factors included limited high-quality evidence, small sample sizes, lack of standardized definitions, heterogeneity in interventions, and short follow-up durations.

**Table 3:** Factors contributing to knowledge gaps regarding weight recurrence, partial and nonresponse to metabolic/bariatric surgery

Factors	Example
Low Quality of Literature	Poor study design
	Small sample size
	Lack or variability in definition of weight recurrence or partial/nonresponse
Heterogeneity of Studies	Mixture of different surgical procedures
	Lack of division between surgical and non-surgical therapy
Follow up	Short follow up duration
	Few subjects followed to longer intervals

## Discussion

This review highlights the substantial variability in the definition of weight recurrence (WR) following metabolic and bariatric surgery (MBS), consistent with prior literature [14]. Many studies failed to distinguish WR from partial or nonresponse—an important oversight given that these entities represent distinct pathophysiologic processes requiring different clinical approaches. Metrics used to define WR varied considerably, including nadir weight, excess weight loss (EWL), total weight loss (TWL), and percent maximum weight loss. Despite this heterogeneity, over half of the studies outlined treatment strategies without a clearly defined threshold for identifying WR, leaving classification to provider discretion and contributing to inconsistent management approaches.

Treatment recommendations varied by provider specialty. Surgeons more often supported endoscopic or surgical revision, while dietitians and nonsurgical providers emphasized behavioral, dietary, and pharmacologic interventions. Few studies offered an integrated treatment pathway. Only 3 studies (2.5%) proposed management algorithms, none of which achieved consensus [18, 29, 33]. This further underscores the need for a unified, evidence-based framework to guide clinical care.

Several studies employed the term “failure” in describing inadequate weight loss outcomes [22, 31, 34]. This terminology carries negative connotations and inappropriately suggests patient culpability. In reality, WR, partial response, and nonresponse likely reflect the chronic and biologically heterogeneous nature of obesity. These outcomes may result from disease-related factors rather than nonadherence or surgical inadequacy. Accordingly, some patients may require adjuvant therapies over time, consistent with a chronic disease management model. Understanding and recognizing WR, partial response, and nonresponse are essential for determining the appropriate timing and modality of additional interventions.

Long-term objectives in MBS include optimizing safety and maximizing weight maintenance. However, variability in defining WR, partial, and nonresponse impedes the development of standardized reporting practices and benchmarks for care [35]. Without consistent terminology, evaluating outcomes, comparing interventions, and initiating timely adjuvant therapy becomes challenging.

We propose standardized definitions for these three entities, as follows [36]:

- **Partial response**: 10–20% total weight loss (TWL).
- **Nonresponse**: <10% TWL.
- **Clinically significant WR**: >20% regain from maximum weight loss.

These definitions use maximum percent TWL as a standardized metric:

- **Maximum %TWL** = ((preoperative weight − nadir weight)/preoperative weight) × 100.
- **% Maximum weight regained** = ((post-nadir weight − nadir weight)/(preoperative weight − nadir weight)) × 100.

These definitions are designed to be clinically applicable, reproducible, and anchored in established benchmarks for weight loss efficacy. By adopting consistent nomenclature, clinicians can more reliably identify patients who require additional support and tailor management accordingly.

### Postoperative Clinical Care Recommendations

Close and consistent follow-up is essential for early identification and intervention in patients experiencing WR, partial response, or nonresponse. We propose the following recommendations to guide postoperative care:

### Routine Postoperative Follow-up

Multiple studies have demonstrated the importance of regular follow-up in detecting deviations from expected postoperative weight trajectories [37–39]. Early visits are critical for identifying complications and evaluating early weight loss patterns, which may predict long-term outcomes [40, 41]. Manning et al. found that weight loss velocity at 3–6 months independently predicted maximal weight loss [42]. Weight loss plateaus often occur between 9 and 12 months postoperatively [43–45], making this window particularly important for identifying partial or nonresponders. While most protocols include 6- and 12-month visits, we recommend an additional follow-up at 9–12 months to improve detection.

### Registered Dietitian (RD) Visits

Rapid weight loss, dietary restriction, and anatomical changes following MBS necessitate RD involvement to ensure adequate nutritional intake and identify maladaptive eating behaviors. Nutrition is one of the most common patient-perceived contributors to suboptimal weight loss [46, 47]. RDs play a critical role in detecting deviations from expected trajectories and initiating early interventions [34, 48–50].

In the absence of anatomic abnormalities (e.g., gastro-gastric fistula, dilated pouch, or gastrojejunal stoma), WR is often associated with sedentary behavior, stress, depression, or neurohormonal changes [40, 49]. These factors can trigger increased appetite, food cravings, and relapse into maladaptive eating patterns. We recommend at least one RD visit at 4–6 weeks postoperatively, with additional visits as indicated by clinical course or behavioral assessment.

### Behavioral Health Resources

MBS entails profound lifestyle changes that affect psychological well-being and eating behavior. Depression, disordered eating, and body image disturbances are prevalent among MBS patients [51]. Maladaptive eating behaviors are strongly associated with suboptimal weight outcomes [34, 48, 50, 52].

Behavioral health specialists can support patients through cognitive-behavioral therapy and behavior change strategies. Evidence suggests that patients may respond more favorably to behavioral interventions postoperatively than preoperatively [53]. Additionally, patients with psychiatric histories or surgical complications are more likely to require mental health services [54]. Given the absence of formal guidelines and frequent insurance limitations, we recommend at least one behavioral health evaluation between 3 and 6 months postoperatively, with further visits based on individual need.

### Physical Activity

While physical activity provides well-documented cardiovascular and metabolic benefits, its direct impact on weight loss following MBS is modest [55, 56]. Exercise may contribute to improved weight maintenance, though data remain mixed [56–62]. Some studies have shown lower WR rates among patients participating in supervised exercise programs [62].

Importantly, lean body mass (LBM) is a major determinant of basal metabolic rate. MBS-related weight loss can result in 20–40% loss of LBM, especially within the first 3 months postoperatively [63–65]. Resistance training has been shown to mitigate LBM loss without compromising overall weight reduction [66]. We recommend all patients engage in structured physical activity at least 3–5 days per week, including resistance-based training. Activity should be individualized based on functional capacity and supervised by physical therapists when appropriate.

### Support Group Participation

Support groups consistently improve postoperative outcomes. Several studies demonstrate a dose-dependent relationship between support group attendance and sustained weight loss [67–70]. Peer support enhances adherence, reinforces behavioral change, and mitigates feelings of isolation. We recommend that patients engage in structured support groups, particularly in the early postoperative period. These groups may be affiliated with bariatric centers or accessed online.

### Management Recommendations for Partial vs. Complete Nonresponders To MBS

**Fig. 1:**
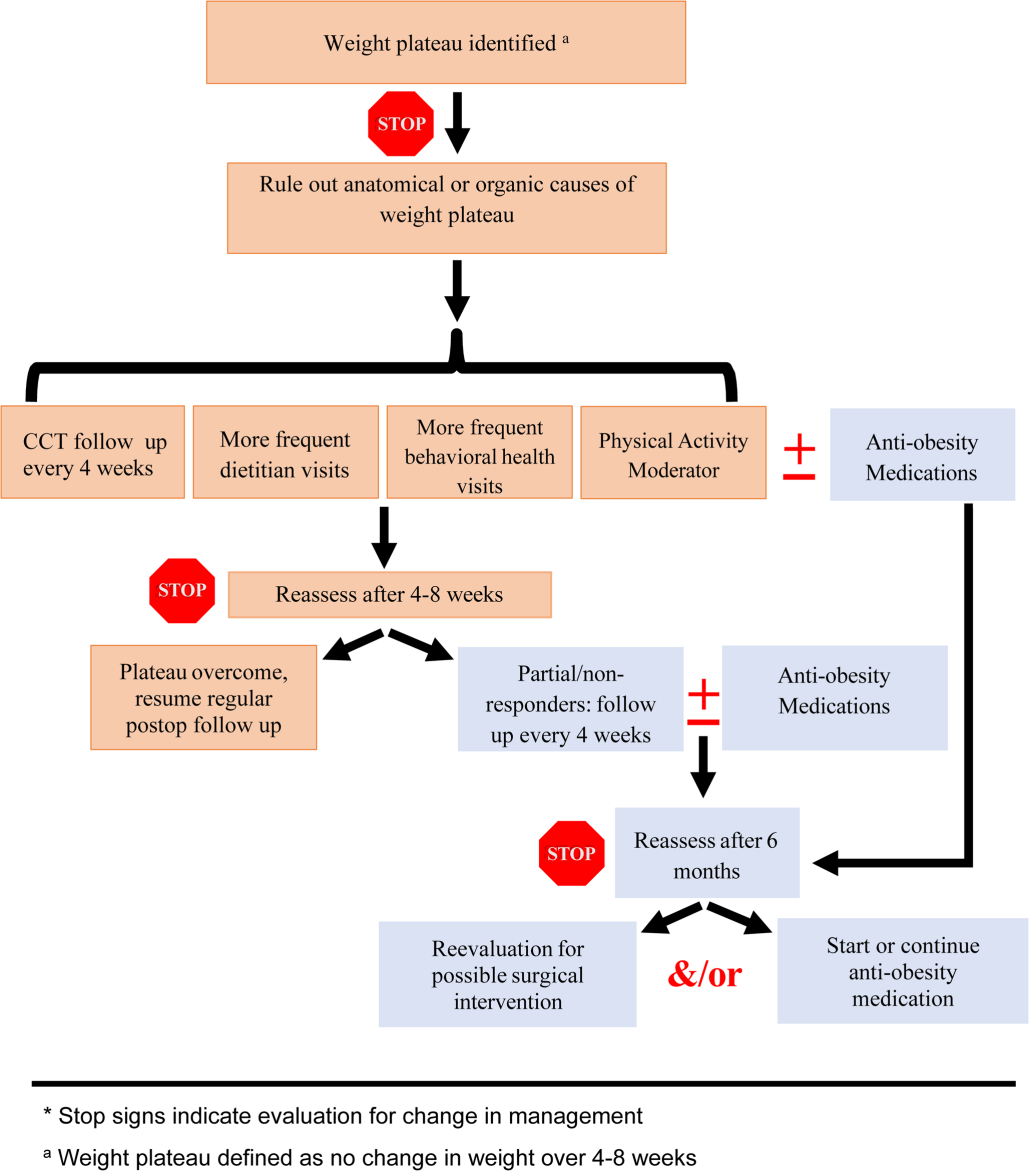
Management of partial and nonresponders to metabolic and bariatric surgery

Partial responders (10–20% total weight loss [TWL]) and nonresponders (< 10% TWL) are typically identified early in the postoperative course. As previously noted, weight plateaus—defined as no change in weight over 4–8 weeks—are commonly observed between 9 and 12 months postoperatively [43–45]. This period is critical for recognizing deviations from expected weight loss trajectories. Figure 1 outlines a management algorithm for these patients.

The first major clinical checkpoint occurs at the time a weight plateau is identified. At this point, the clinical care team (CCT) should re-evaluate the patient’s health status, behaviors, and weight trajectory. We recommend increasing CCT visits to every 4 weeks. Enhanced frequency of dietitian and behavioral health visits should also be considered to assess nutritional adequacy, adherence to dietary guidelines, and presence of disordered eating. Frequency of these ancillary visits should be determined based on clinical need and resource availability (e.g., insurance coverage). When gaps in access to these services exist, the CCT should assume responsibility for interim support. Physical activity patterns and support group participation should also be re-evaluated and adjusted as needed. In particular, addressing weight plateaus—often a source of patient distress—through peer support may alleviate discouragement and promote continued engagement [67].

Adjuvant therapy decisions should be individualized. In cases where it is unclear whether a patient is experiencing partial or nonresponse, close follow-up with reinforcement of nutrition, behavioral interventions, and physical activity may be sufficient to restore expected weight loss. For clearly identified partial or nonresponders, pharmacologic or surgical interventions may be warranted. Options include anti-obesity medications (AOMs), endoscopic procedures, or revisional bariatric surgery.

The second checkpoint should occur no more than 12 weeks after the initial plateau is identified [71]. Patients who resume weight loss may transition to routine or modified follow-up based on institutional protocols. Patients with continued suboptimal response should be formally classified as partial or nonresponders and considered for intensified therapy. These individuals require ongoing, frequent assessments by the multidisciplinary team.

In our practice, AOMs serve as first-line adjuvant therapy when anatomical abnormalities are excluded [71, 72]. Although glucagon-like peptide-1 (GLP-1) receptor agonists are widely used, alternative agents should be considered when contraindicated or ineffective [72]. Patients initiated on pharmacotherapy should remain on treatment for a minimum of 6 months [71, 73, 74]. During this period, medication dose and combinations should be regularly adjusted at 4-week follow-ups. Patients with favorable responses and no contraindications may continue long-term therapy. Those without meaningful benefit, or who prefer not to continue medications, should be evaluated for surgical interventions—including endoluminal or operative revisions—as appropriate. Surgical options may also be considered before or in combination with pharmacotherapy, depending on clinical factors and patient preference.

### Management Recommendations for Weight Recurrence Following MBS

**Fig. 2:**
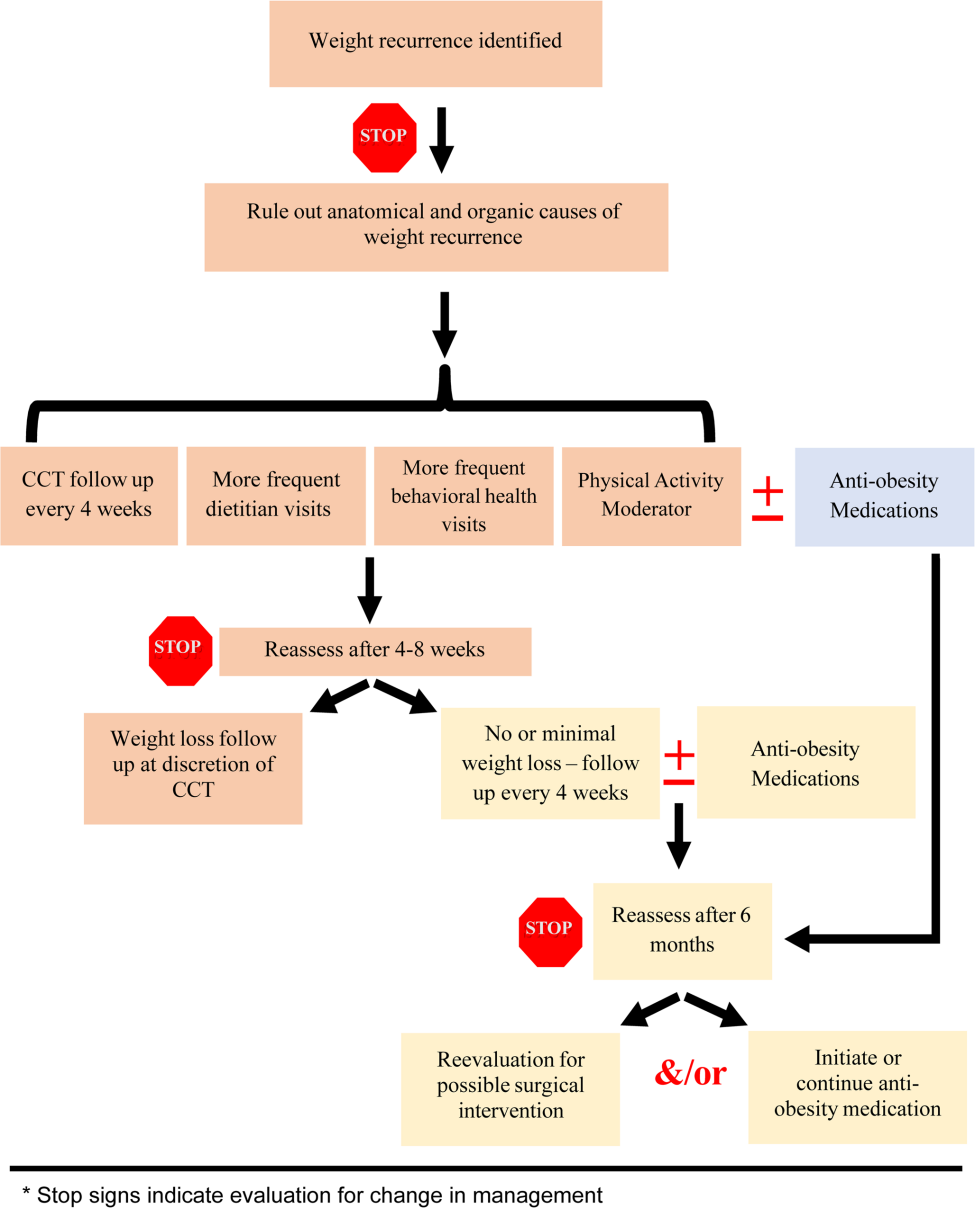
Management of patients with weight recurrence following metabolic and bariatric surgery

Unlike partial or nonresponders, patients with weight recurrence (WR) typically follow an appropriate early postoperative weight loss trajectory but then experience weight gain after reaching their nadir. These patients are usually identified beyond 12–18 months postoperatively. Figure 2 outlines our recommended management algorithm for WR.

Once WR is identified, patients should undergo frequent and structured follow-up to assess causative factors and monitor progression. We recommend clinical care team (CCT) visits every 4 weeks during the initial intervention phase. Additional visits with registered dietitians (RDs) and behavioral health providers are encouraged to identify maladaptive eating patterns, screen for psychosocial stressors, and guide lifestyle interventions. While frequency may vary by clinical need and resource availability, we suggest at least two RD and behavioral health visits within the first two months of WR detection.

Given that initial weight loss may include significant lean body mass (LBM) reduction, and subsequent WR is typically characterized by fat mass regain, these patients often exhibit a lower basal metabolic rate than before surgery [63]. Thus, assessment and optimization of physical activity are essential. Exercise regimens should emphasize LBM preservation and metabolic support. Additionally, clinicians should evaluate for organic contributors to WR, including thyroid dysfunction or, in select cases, genetic syndromes.

Adjuvant therapy should be considered no later than 8 weeks following identification of WR. Treatment options—whether surgical or nonsurgical—should be tailored to patient-specific factors and initiated at the discretion of the provider. In our clinical experience, first-line therapy typically involves anti-obesity medications (AOMs) in the absence of anatomical abnormalities. For patients with anatomical contributors (e.g., gastro-gastric fistula, pouch dilation), endoscopic or surgical revision may be prioritized.

As with nonresponders, patients starting AOMs should remain on therapy for at least 6 months. During this period, clinicians should assess treatment efficacy, adjust dosing, and consider combination pharmacotherapy as needed. For patients demonstrating a favorable response and a willingness to remain on lifelong therapy, continued use of AOMs is appropriate [75, 76]. For those with inadequate response or those declining medical management, revisional surgical options should be discussed and pursued when indicated. Given the chronic, relapsing nature of obesity, a multimodal approach—integrating surgical and medical therapies—may be necessary for durable success.

### Future Directions

In addition to improving clinical care, standardized definitions for weight recurrence (WR), partial response, and nonresponse are essential for advancing research. The heterogeneity observed in our review illustrates the lack of consistency in the literature. While standardized reporting guidelines exist for weight loss outcomes, comorbidity resolution, and follow-up, no such consensus currently addresses WR, partial response, or nonresponse [35]. We propose that our definitions serve as a foundation for developing uniform study designs and more consistent outcome reporting. Such efforts would enable the collection of high-quality data to inform best practices and drive clinical innovation.

The national Metabolic and Bariatric Surgery Accreditation and Quality Improvement Program (MBSAQIP) database remains a powerful resource for tracking outcomes and informing care at scale. Incorporating standardized definitions into MBSAQIP would allow for national benchmarking of WR, partial response, and nonresponse rates, as well as identification of trends in treatment strategies and their timing. In addition, the MBSAQIP Surgical Risk/Benefit Calculator, which currently estimates expected weight loss at 12 months postoperatively, could be enhanced through these definitions. By comparing observed versus expected weight trajectories—both at the individual and national level—clinicians would be able to identify deviations earlier and initiate timely interventions. Patients, too, would benefit from visual tools that illustrate their weight response and offer context for treatment decisions.

Ultimately, identifying patterns of weight loss and regain using standardized definitions may reveal predisposing factors that inform preventive strategies and optimize long-term outcomes.

## Conclusion

Weight recurrence (WR), partial response, and nonresponse following metabolic and bariatric surgery (MBS) likely represent manifestations of the chronic, relapsing nature of obesity rather than failures of surgical intervention. As such, adjuvant therapy may constitute an expected component of long-term obesity management. Current variability in the definitions of these terms impairs outcome assessment, obscures the true prevalence of these conditions, and hinders the development of effective treatment strategies.

We propose using percent maximum total weight loss (TWL) to standardize definitions: 10–20% TWL to define partial response, < 10% TWL to define nonresponse, and > 10% regain of maximum weight lost to define WR. These definitions provide a reproducible framework for clinical decision-making.

Using these definitions, we offer practical, evidence-informed recommendations for postoperative surveillance and management. This framework facilitates early detection and timely intervention for patients with WR or partial and nonresponse. We hope these efforts empower clinicians to better support this complex patient population and promote consistency in outcomes reporting.

Looking forward, standardized terminology and clinical algorithms may be leveraged across institutional databases and digital tools to improve identification, communication, and treatment of WR, partial response, and nonresponse—ultimately maximizing the long-term benefits of MBS.

## Data Availability

No datasets were generated or analysed during the current study.
